# Retrospective Evaluation of Nasopalatine Canal Anatomy, Dimensions, and Variations with Alveolar Bone in Patients Scheduled for Maxillary Anterior Dental Implant Surgery Using Cone Beam Computed Tomography

**DOI:** 10.3390/tomography11100114

**Published:** 2025-10-12

**Authors:** Savaş Özarslantürk, Seval Ceylan Şen, Özlem Saraç Atagün

**Affiliations:** 1 Department of Dentomaxillofacial Radiology, Gulhane Faculty of Dentistry, University of Health Sciences, Ankara 06010, Turkey; savas.ozarslanturk@sbu.edu.tr; 2 Department of Periodontology, Gulhane Faculty of Dentistry, University of Health Sciences, Ankara 06010, Turkey; seval.ceylansen@sbu.edu.tr

**Keywords:** anatomical variations, nasopalatine canal, dental implant, anterior maxilla, cone beam computed tomography

## Abstract

**Objective:** This study aimed to retrospectively evaluate the anatomical structure, dimensions, and variations in the nasopalatine canal using cone beam computed tomography (CBCT) in patients undergoing implant treatment in the maxillary anterior region. The goal was to identify potential risks and complications that may arise during surgical procedures. Additionally, canal shape, number, and its relationship with gender and nasal septa were assessed as secondary parameters. **Methods:** This retrospective study included CBCT scans of 185 patients who applied for implant treatment in the anterior maxilla between January 2021 and December 2023. Patients with edentulous anterior maxillae and no pathological lesions in the implant region were included. CBCT images were analyzed in sagittal, axial, and coronal planes using standardized measurement protocols. The shape, number, dimensions, and angulation of the nasopalatine canal were evaluated by two blind observers with high inter-rater agreement. Morphological classifications and canal–implant relationships were recorded as primary and secondary outcome parameters. **Results:** Among the 185 CBCT scans analyzed, the nasopalatine canal was most frequently observed as a single structure (87.6%), typically located in the central incisor region, with a cylindrical morphology in the sagittal plane (44.9%) and a single shape in the coronal plane (52.4%). While no significant differences were found in morphometric parameters by age or sex, accessory canal locations differed significantly between sexes (*p* = 0.040). **Conclusions:** The anatomical characteristics and morphometric measurements of the nasopalatine canal exhibit considerable variability, underscoring the importance of individualized CBCT assessment during implant planning in the anterior maxilla. Recognizing accessory canal positions, particularly their sex-related differences, is critical for minimizing surgical complications and optimizing outcomes.

## 1. Introduction

Dental imaging is a crucial criterion that supports the anamnesis and physical examination in the clinical evaluation and diagnosis of patients in dentistry. Roentgen introduced intraoral radiography in the period following the discovery of X-rays in 1895 [1]. In the following periods, digital imaging, computed tomography (CT), magnetic resonance imaging (MRI), positron emission tomography (PET), and cone beam computed tomography (CBCT) were developed, and three-dimensional imaging of the maxillofacial region became possible.

In general, CBCT offers enormous benefits for implant applications and all surgical treatments in dentistry. Advances in CBCT technologies and innovations in imaging software are leading to improvements in scanning equipment, ultimately allowing physicians to plan more accurate and precise treatment [2]. The most crucial advantage of CBCT is that it enables the practitioner to edit and monitor three-dimensional images in sagittal, axial, and coronal planes in a personal computer environment. Editing and visualizing data in CBCT can be performed on personal computers [3]. Today, thanks to their technology, CBCT devices can achieve pixel resolution of the imaged objects at sub-millimeter levels. With modern CBCT devices, sub-millimetric voxel resolution with an isotropic structure between 0.125 and 0.4 mm can be obtained [4].

The nasopalatine canal (NPC) and incisive foramen, which carry the nasopalatine nerve and artery, represent the major neurovascular bone structures in the anterior maxilla. Dentists are familiar with these anatomical formations and can identify them on conventional radiographs. The anterior superior alveolar (ASA) nerve and artery also reach this region through a bony canal called the canalis sinuous [5]. Anatomical variations (e.g., additional foramina and canals) may be present in the neurovascular structures in the anterior region, and these variations may affect treatment plans and outcomes by creating potential complications for surgical procedures. The anatomy and morphology of the NPC have been studied by many authors using three-dimensional radiographs. In a few studies, canal shape, length, and curvature, as well as specific factors thought to influence canal morphology, such as ethnicity, gender, age, and the presence or absence of teeth, were analyzed [5,6].

This study aimed to retrospectively evaluate the anatomy, dimensions, and variations in the NPC in relation to the alveolar bone using CBCT in patients presenting for dental implant treatment in the maxillary anterior region. The data obtained showed the degree and frequency of complications that may be encountered during surgical procedures in patients treated with dental implants in the maxillary anterior region. Additionally, the shape and numerical variations in the nasopalatine canal, as well as its relationship with the nasal septum, were assessed as secondary outcome parameters according to gender.

## 2. Methods

This retrospective study was approved by the Health Sciences University Gülhane Scientific Research Ethics Committee (2024/237). In this study, the CBCT images of 185 patients who applied to the Department of Periodontology, Gülhane Faculty of Dentistry, University of Health Sciences, between 1 January 2021 and 31 December 2023, with the complaint of edentulousness in the anterior maxilla or who have an indication for immediate implantation were scanned before implant placement. In CBCT imaging, patients who were not edentulous in the anterior maxillary region or who did not have an indication for immediate implantation, as well as those with pathological formations in the implant placement area, were to be excluded from the study. This study is a retrospective analysis of anonymized data. In accordance with the ethics committee approval, no additional patient consent was required for the use of CBCT images due to the retrospective design of the study.

CBCT images were obtained using a 3D Accuitomo 170 (J. Morita Mfg. Corp., Kyoto, Japan) device. The device’s own software program, i-Dixel (i-Dixel 2.0/One Volume Viewer, J. Morita Mfg. Corp., Kyoto, Japan), was used for image acquisition, analysis, and measurements. The nasopalatine canal and maxillary anterior region were examined in the sagittal, frontal, and axial planes with 1 mm slice intervals on CBCT images with a 100 mm diameter and 100 mm height FOV. A Dell^TM^ 3008WFP, 1920 × 1200 pixels, 32-bit, 30″ Flat Panel Monitor (Dell Inc., Round Rock, TX, USA) was also used for the analysis of CBCT images and measurements. Measurements were performed by two independent observers (S.Ö, S.C.Ş) and demonstrated strong inter-rater reliability (Pearson’s r = 0.92). The average measurements obtained by the two observers were calculated and used in the analysis.

In the coronal sections, each section was oriented so that the palatal plane was horizontally aligned. In contrast, in the axial sections, the sections were planned perpendicular to the maxillary anterior process for the analysis of the regions of interest, i.e., the anterior teeth. For the measurements, the midpoint of the future implant site, 5 mm from the distal aspect of the adjacent lateral tooth, and 5 mm from the mesial aspect of the adjacent lateral tooth were selected as the measurement sites. To perform the measurements, all alveolar prominences and relevant anatomical structures in the anterior maxilla (e.g., spina nasalis anterior) were imaged, each as the most significant respective image.

The crest width of the orofacial bone was measured in coronal slices perpendicular to the alveolar ridge. The measurements were taken 2 mm from the apex of the alveolar crest to obtain more representative values and to compensate for the convex configuration of the crest.

**Figure 1:**
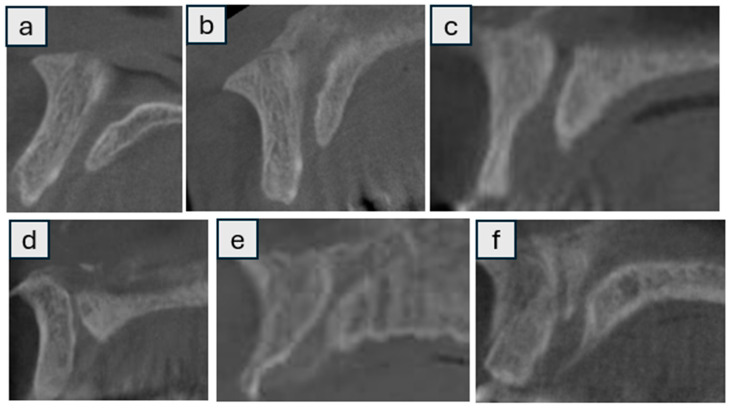
Shapes of nasopalatine canals in the sagittal plane: cylindrical (**a**), banana (**b**), funnel (**c**), cone (**d**), hourglass (**e**), tree branch (**f**).

**Figure 2:**
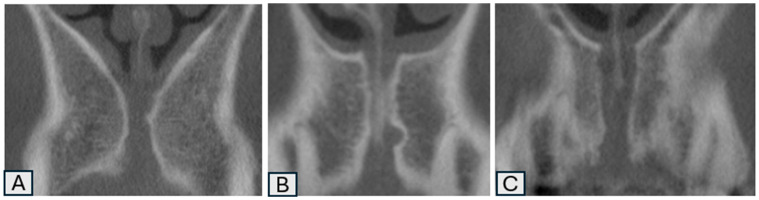
Shapes of nasopalatine canals in the coronal plane: single (**A**), two parallel (**B**), and Y-shaped (**C**).

**Figure 3:**
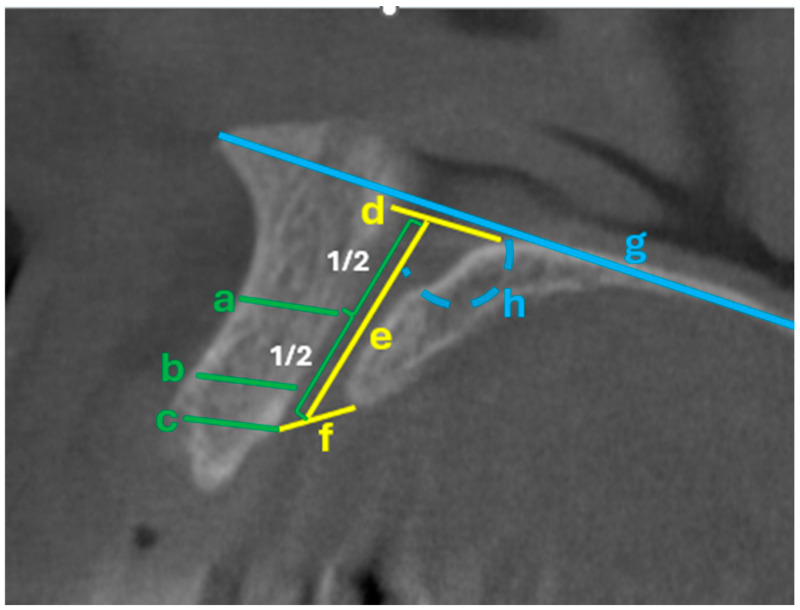
The following landmarks were selected for analysis of the sagittal CBCT images [all measurements in mm (except h; h shows the angle in degrees)]; (a) cranial reading evaluates the distance from the buccal border in the middle of the nasopalatine canal to the facial aspect of the buccal bone wall. (b) Distance midway from the buccal bone wall of the nasopalatine canal to the facial aspect of the bone wall using a horizontal line from the palatal border of the incisive foramen. (c) Crestal distance from the buccal border of the incisive foramen to the facial aspect of the buccal bone plate. (d) The diameter of the nasopalatine foramen. (e) The length of the nasopalatine canal. (f) The diameter of the incisive foramen. (g) Palatal plane. (h) Angle formed by the nasopalatine canal and the palatal plane.

**Figure 4:**
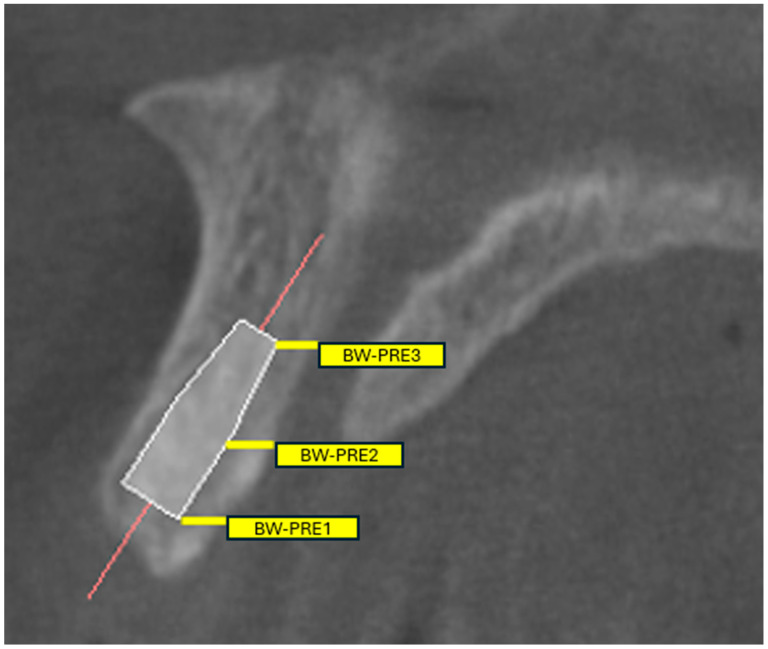
Pre-implant placement measurements. The angulation of the alveolar ridge and the horizontal distance between the palatal-most aspect of the root to the nasopalatine canal were recorded as Bw-pre 1, Bw-pre 2, and Bw-pre 3 for the apical (coronal), middle, and nasal (palatal) levels, respectively.

The shape, number, size, morphology, and variations in the NPC were analyzed by scanning sagittal and coronal CBCT slices (Figure 1 and Figure 2) [7] categorized into nine groups according to whether the shape was cylindrical, banana, funnel, cone, hourglass, and tree branch (sagittal plane) (Figure 1), single, two parallel, and Y-shaped (coronal plane) (Figure 2) [8]. Supplementary Table S1 provides an explicit correspondence between the present study’s 9-category morphology scheme and the traditional 4-category and 2-category schemes, facilitating comparative research and meta-analytic synthesis. In addition, the width (diameter) and length of the NPC, as well as the distance between the canal and the buccal bone (at three points: apical, middle, and nasal), were recorded numerically (in millimeters) as secondary outcome parameters. (Figure 3) In cases where there was more than one nasopalatine canal, the tooth region and the number of teeth were recorded. Finally, the apical (buccal), midcoronal (middle), and nasal (palatal) angles of the nasopalatine canal with the implant socket were recorded numerically (in millimeters) in terms of the position of the implant planned to be placed in the maxillary anterior region (Figure 4) [9,10].

### Operational Metrics for Pre-Operative Risk Appraisal

For clinical translation and guided-surgery planning, we pre-specified the reporting of (i) the shortest NPC–central incisor root distance at apical/middle/nasal levels (Bw-pre 1/2/3), (ii) NPC diameters at nasal and palatal openings, (iii) NPC length, (iv) buccal bone thickness anterior to the canal at three levels, and (v) Angle 1 (NPC–palatal plane). In the Results, these are summarized with means and distribution cut-points (quartiles) to enable threshold-based planning (e.g., flagging values in the lowest quartile as “heightened-risk proximity”). Where applicable, sex-stratified summaries are provided to reflect the observed variation in accessory canal location.

## 3. Statistical Analysis

The sample size for this study, which aimed to investigate the morphometric and morphological characteristics of the NPC and its surrounding anatomical structures, was calculated to achieve a statistical power of at least 80% and a Type I error rate of 5% for each variable. Guided by published CBCT data showing sex-related differences in NPC dimensions, we adopted a conservative standardized mean difference in d = 0.41 to ensure adequate power across multiple morphometric endpoints. Under these parameters, the required total sample was *n* ≈ 184. To offset potential exclusions due to image artifacts or incomplete visualization, we specified a target of *n* = 185 [10]. The normality of distribution for continuous variables was assessed using the Kolmogorov–Smirnov test and Skewness–Kurtosis values. As the data were normally distributed, parametric tests were employed.

Descriptive statistics for the variables were expressed as mean, standard deviation, number (n), and percentage (%). For comparisons of continuous variables between groups, the Independent Samples *t*-test and the One-Way Analysis of Variance (ANOVA) were used. Post hoc comparisons following ANOVA were performed using the Duncan test. The relationships between categorical variables were analyzed using the Chi-square test, while correlations between continuous variables were evaluated using Pearson correlation coefficients.

The significance level of *p* < 0.05 was considered statistically significant. All analyses were performed using the SPSS statistical software package (IBM SPSS for Windows, version 26).

## 4. Results

A total of 185 CBCT scans were analyzed in this study. The mean age of the patients was 47.94 ± 13.61 years, with 50.3% of the patients being male and 49.7% female (Table 1). A single NPC was observed in most cases (87.6%), while 12.4% had two canals. The most common location of the canal was in the central incisors’ region (54.6%). When an accessory canal was present, it was most often situated between the central and lateral incisors (47.8%) or in the lateral incisors’ region (43.5%). The most frequently observed NPC morphology was cylindrical in the sagittal plane (44.9%) and single in the coronal plane (52.4%) (Table 1).

Morphometric analysis revealed that the mean nasal opening diameter of the NPC was 3.15 ± 0.66 mm, while the palatal opening measured 3.42 ± 0.62 mm. The average canal length was 11.39 ± 1.98 mm. The mean buccal bone thickness anterior to the canal was 9.70 ± 1.58 mm at the coronal point, 7.10 ± 1.04 mm at the midpoint, and 9.61 ± 0.54 mm at the apical point. The mean angle between the canal and the horizontal plane was 107.50 ± 4.43°, and the angle with the central incisor was 9.26 ± 3.48°. The shortest distance from the oral foramen of the canal to the palatal root surface of the central incisors was 1.66 ± 0.45 mm (Table 2).

When stratified by age groups (20–39, 40–59, and 60–75 years), no statistically significant differences were found in any morphometric measurements, including canal diameter, length, angles, or buccal bone thickness (*p* > 0.05 for all) (Table 3).

Sex-based comparisons showed no significant differences in any of the continuous anatomical parameters between males and females (*p* > 0.05) (Table 4). Although the angle between the NPC and the horizontal plate (Angle 1) was slightly greater in males (108.11°) than in females (106.89°), the difference did not reach statistical significance (*p* = 0.061) (Table 4). Similarly, categorical anatomical characteristics, such as the number, location, and shape of the canal, did not differ significantly between age groups (Table 5).

To facilitate chairside use, we additionally present quartiles for the NPC–root distances (Bw-pre 1/2/3) and buccal bone thicknesses so that cases falling into the lowest quartile can be flagged for modified implant angulation, narrower/shorter implant selection, or augmentation during guided surgery. (For context, in this cohort the mean NPC length was 11.39 ± 1.98 mm, buccal bone thickness anterior to the canal was 9.70 ± 1.58/7.10 ± 1.04/9.61 ± 0.54 mm at coronal/middle/apical levels, and the shortest oral-foramen–to–palatal root distance (Bw-pre 1) was 1.66 ± 0.45 mm; Angle 1 averaged 107.50 ± 4.43°).

The distribution of most categorical anatomical features, including canal number, central location, and morphology, did not differ significantly by sex. However, the area of accessory canals showed a significant sex-related difference (*p* = 0.040). Accessory canals were more likely to be positioned near the midline in males, while in females, they tended to be located more laterally (Table 6).

## 5. Discussion

This retrospective study examined the anatomical features, morphometric measures, and alterations of the NPC concerning the adjacent alveolar bone utilizing CBCT in patients preparing for dental implant insertion in the maxillary anterior region. The findings offer essential insights into possible anatomical issues that may occur during surgical treatments in this area, especially with the possibility of NPC injury. Given the proximity of the nasopalatine canal to planned implant sites, a thorough understanding of its variations, including shape, number, and its spatial relationship to the nasal septum, is essential for optimal surgical planning. To further understand these findings’ clinical significance and practical ramifications, they are examined within the framework of recent research.

López Jornet et al. [10], in their study investigating the characterization of the anterior palatal region, reported that males had significantly greater NPC dimensions and alveolar bone thickness anterior to the canal compared to females. A CBCT-based systematic review assessing sex and population variations reported that nasopalatine canal length and the diameters of the incisive and Stenson foramina are generally larger in males than females, and that NPC dimensions vary across different populations [11]. Similarly, Soman reported that males had a statistically longer canal (13.58 mm) compared to females (11.91 mm) [12]. In this study, no statistically significant differences were found between male and female patients in any of the anatomical measurements examined (*p* ≥ 0.05). However, the angle formed between the nasopalatine canal and the palatal plane (Angle 1) did not reach statistical significance (*p* = 0.061) and should be interpreted cautiously; further investigation is warranted. Although not reaching the conventional threshold for significance, the mean angle in males (108.11°) appeared slightly larger than in females (106.89°). This numerical difference, although modest, may suggest a trend that could become significant in studies with larger sample sizes. Therefore, this finding may indicate a weak signal of potential anatomical variation between sexes that warrants further investigation with more robust statistical power.

Alkanderi et al., in their study evaluating the incidence of NPC perforation, found that NPC perforation occurred in only 8% of cases, and that the risk of perforation was significantly associated with the palatal bone width of the central incisors and the angulation of the nasopalatine canal, indicating that narrower palatal bone and greater canal angulation increased the likelihood of perforation [9]. De Oliveira-Santos et al., in their study investigating neurovascular anatomical variations in the anterior palate using CBCT, reported that over 15% of the population exhibited additional foramina measuring between 1 and 1.9 mm in diameter, most of which were located in variable positions and associated with bony canals extending either as continuations of the canalis sinuous or toward the floor of the nasal cavity [7]. In terms of anatomical variations, this study found that most patients had a single nasopalatine canal (NPC), mostly located in the midline (central incisor region); when multiple canals were present, the accessory canal was typically positioned more laterally, and the most common canal shapes (sagittal) were cylindrical and cone. These results emphasize how crucial it is to understand the anatomical heterogeneity in the nasopalatine region to ensure safe and efficient surgical planning, especially when the anterior maxilla is involved.

In a study conducted on partially edentulous patients, it was reported that individuals with central incisor loss of more than one year exhibited thinning of the coronal portion of the buccal bone wall. That age was significantly associated only with the length of the nasopalatine canal, which tended to decrease with increasing age [8]. However, Soman reported no significant differences in NPC length across age groups [12]. Similarly, in this study, no statistically significant differences were found between the age groups in any of the anatomical measurements examined (*p* ≥ 0.05). Moreover, no statistically significant differences were observed between age groups in any of the evaluated nasopalatine canal characteristics, including the number, position, presence of accessory canals, or shape of the canals (*p* ≥ 0.05). However, the only significant difference observed was in the position of the accessory canal when multiple canals were present, with a tendency for the accessory canal to be closer to the midline in males and more laterally positioned in females (*p* = 0.040). Individual anatomical variability, compensatory bone remodeling, or the small sample size within each age group, which would have hindered the detection of minor age-related alterations, could all be responsible for the absence of significant differences.

In a study evaluating the outcomes of virtual implant placement in the anterior maxillary region, implant positioning based on the cingulum emergence plan was associated with a lower likelihood of invasion of the nasopalatine canal compared to the incisal edge emergence plan [13]. These findings emphasize the importance of considering local anatomical structures, particularly the trajectory and proximity of the nasopalatine canal, during immediate implant planning. Similarly, in our study, the NPC was found to have an average length of 11.39 mm and a mean diameter of approximately 3.3 mm, typically widening slightly toward the palatal opening. The thinnest part of the buccal bone anterior to the canal was observed at its midpoint (mean 7.10 mm). Additionally, the canal was inclined antero-inferiorly relative to the horizontal plane (mean angle 107.5°), and most importantly, its oral opening was located close to the roots of the central incisors (mean distance 1.66 mm). According to these results, CBCT-based personalized anatomical evaluation is crucial for reducing problems and improving surgical results in the anterior maxilla.

Although several studies have attempted to classify the NPC morphology, a universally accepted classification system has not yet been established. For example, Mardinger et al. and Guncu et al. categorized NPC shapes into four types: hourglass, funnel, banana, and cylindrical, based on sagittal CBCT sections [14,15]. In contrast, Liang et al. proposed a simpler classification with only two types: conical and cylindrical [6]. In contrast to previous studies, the present study employed a more comprehensive classification system, identifying nine distinct NPC morphologies in sagittal and coronal sections: cylindrical, conical, funnel, banana, hourglass, and tree-branch in the sagittal plane; and single, two-parallel, and Y-shaped configurations in the coronal plane. This approach offers a more comprehensive understanding of anatomical variability in the anterior maxilla. Öçbe et al., using a multimodal imaging approach combining CT and magnetic resonance imaging (MRI), reported that 63.01% of nasopalatine canals were round or oval in the axial plane, while 47.69% exhibited a Y-shaped configuration in the coronal plane; furthermore, they found that patients with anterior tooth loss had a significantly larger incisive foramen diameter (*p* < 0.001), likely due to bone remodeling following tooth extraction [16]. Firincioglulari et al., in their study evaluating the morphological variations in the nasopalatine canal using CBCT, found a significant relationship between the shape of the NPC and the horizontal dimensions of the anterior maxilla, reporting reduced horizontal bone widths in the premaxilla for banana- and funnel-shaped NPCs. Additionally, they observed that individuals with hourglass-shaped NPCs exhibited a significantly larger anteroposterior diameter of the nasal foramen compared to all other shapes [17]. A CBCT study analyzing 360 scans reported a mean nasopalatine canal length of 12.51 mm, with the hourglass shape being the most common configuration in both males (80.62%) and females (87.01%) [12]. Similarly, Etoz and Sisman reported that the hourglass-shaped nasopalatine canal was the most frequently observed morphology (38.78%), followed by the funnel-shaped type, which accounted for 27.35% of the cases [18]. However, Milanovic et al. reported that the funnel-shaped nasopalatine canal had the highest incidence (35%), and that the banana-shaped canal was associated with a general reduction in anterior maxillary dimensions compared to the other canal types [19]. In the sagittal section, the most observed nasopalatine canal shape was cylindrical (44.9%, *n* = 83), followed by the conical type (25.4%, *n* = 47). Less frequent morphological variations included the hourglass shape (4.3%), tree-branch-like shape (1.6%), and banana shape (1.6%).

To summarize, these findings underscore the considerable variability in NPC morphology reported across studies and highlight the importance of using detailed classification systems to better understand anatomical differences relevant to clinical practice.

Furthermore, to strengthen the clinical relevance of the present findings, it is essential to emphasize their implications for implant dentistry. The morphometric assessment of the nasopalatine canal obtained from CBCT scans provides valuable guidance for implant planning in the anterior maxilla. Variations in canal morphology and accessory canal positioning should be carefully considered to minimize surgical complications, particularly the risk of neurovascular injury or implant failure. Translating these radiological findings into clinical decision-making underscores the importance of individualized preoperative evaluation, which contributes to safer implant placement and enhanced long-term treatment outcomes.

This study has some limitations. Due to its observational design, the study is limited in its capacity to infer causality, as it can reveal associations between variables but cannot conclusively determine cause-and-effect relationships. Another limitation is the recruitment of participants from a single geographic region, which may limit the generalizability of the findings, as anatomical variations in the nasopalatine canal could differ across diverse populations. This study did not account for the duration since tooth extraction or the potential effects of alveolar bone resorption, which should be considered as additional limitations when interpreting the findings. The findings of this study highlight the need for further well-designed clinical research incorporating adequate and reproducible assessment methods to investigate the NPC.

### Clinical Implications for Guided Surgery and Pre-Operative Assessment

Our CBCT-based characterization of the nasopalatine canal (NPC) supports a practical, stepwise workflow for anterior maxillary implant planning:

1. **Screen morphology and multiplicity:** Note cylindrical versus conical forms and the presence of accessory canals. In our cohort, the location of the accessory canal differed by sex (*p* = 0.040); therefore, clinicians should inspect nearer the midline in males and more laterally in females.
2. **Quantify proximity:** Report Bw-pre 1/2/3 values and flag the lowest quartile—or a validated conservative threshold—as indicating heightened-risk proximity. Previous morphometric studies have demonstrated that the mean bone width between the central incisor root and the NPC ranges from approximately 1.4 mm at the coronal third to 3.2 mm at the apical third [9,10]. Accordingly, the risk of NPC perforation becomes clinically significant when the distance between the canal and the planned implant trajectory is less than 2 mm.
3. **Verify buccal bone corridor:** If mid-level buccal thickness is low, anticipate the need for angulation adjustment, narrower or shorter implants, or minor site development.
4. **Assess canal trajectory (Angle 1):** Markedly oblique canals reduce the safe implant window; consider a more palatal entry point or a custom sleeve offset to prevent encroachment.
5. **Translate to guide design:** Overlay the main and accessory canals, enforce a minimum clearance of ≥2 mm, and lock sleeves to maintain the planned prosthetically driven corridor.
6. **Plan alternatives:** Predefine backup implant diameter, length, and angulation options—or staged augmentation protocols—when canal proximity is high.
7. **Inform consent and monitor:** Document the elevated risk when below the safety threshold and monitor for early signs of nasopalatine bundle irritation, such as paresthesia or intraoperative bleeding.

These steps translate anatomical measurements into a concise, reproducible checklist that enhances the predictability of guided implant surgery, minimizes the risk of NPC encroachment, and optimizes both esthetic and functional outcomes.

## 6. Conclusions

This study revealed that the nasopalatine canal most commonly appears as a single, straight, or Y-shaped structure located in the central incisor region. Although most anatomical parameters did not differ significantly by age or sex, accessory canal positions showed sex-related variation. These findings underscore the importance of individualized CBCT assessment in preventing surgical complications and ensuring optimal implant planning in the anterior maxilla.

## Figures and Tables

**Table 1:** Descriptive statistics of demographic variables and nasopalatine canal characteristics of the patients (*n* = 185).

		N	%
**Age**	20–39 age	62	33.5%
	40–59 age	75	40.5%
	60–75 age	48	25.9%
**Gender**	Man	93	50.3%
	Female	92	49.7%
**Number of nasopalatine canal (NPC)**	1	162	87.6%
	2	23	12.4%
**The region where the nasopalatine canal is located**	Central incisor region	101	54.6%
	Between central and lateral	64	34.6%
	Lateral incisor region	18	9.7%
	Canine region	2	1.1%
**Additional foramina observed in the palate according to their** **location**	Between central and lateral	11	47.8%
	Lateral incisor region	10	43.5%
	Canine region	2	8.7%
**Morphology of the nasopalatine** **canal (Sagittal)**	Cylindrical	83	44.9%
	Cone	47	25.4%
	Funnel	41	22.1%
	Banana-shaped	3	1.6%
	Hourglass-shaped	8	4.3%
	Tree branch	3	1.6%
**Morphology of the nasopalatine** **canal (Coronal)**	Single	97	52.4%
	2 parallel	23	12.4%
	Y-shaped	65	35.1%

**Table 2:** Descriptive statistics of continuous, categorical, and anatomical measurements of the patients (*n* = 185).

		Mean	Std. Dev.
**Age**		47.94	13.61
**Canal diameter (mm)**	Nasal	3.15	0.66
	Palatal	3.42	0.62
	Mean	3.29	0.45
**Canal length (mm)**		11.39	1.98
**Buccal anterior to the canal (mm)**	Coronal	9.70	1.58
**Buccal anterior to the canal (mm)**	Middle	7.10	1.04
**Buccal anterior to the canal (mm)**	Apical	9.61	0.54
**Angle 1 (with horizontal plate)**		107.50	4.43
**Angle 2 (with palatal bone margin)**		6.35	1.81
**Angle 3 (with incisor)**		9.26	3.48
**Bw-pre 1 (mm)**		1.66	0.45
**Bw-pre 2 (mm)**		2.53	0.58
**Bw-pre 3 (mm)**		3.59	0.82

The angulation of the alveolar ridge and the horizontal distance between the palatal-most aspect of the root to the nasopalatine canal were recorded as Bw-pre 1, Bw-pre 2, and Bw-pre 3 for the apical (coronal), middle, and nasal (palatal) levels, respectively.

**Table 3:** Comparison of various anatomical measurements according to age groups (*n* = 185).

		Age						
		20–39 Age		40–59 Age		60–75 Age		
		Mean	SD	Mean	SD	Mean	SD	* *p.*
**Canal diameter (mm)**	Nasal	3.26	0.61	3.02	0.68	3.22	0.66	0.071
	Palatal	3.43	0.66	3.40	0.61	3.43	0.61	0.940
	Mean	3.35	0.49	3.21	0.43	3.33	0.43	0.162
**Canal length (mm)**		11.51	2.16	11.44	1.79	11.17	2.03	0.645
**Buccal anterior to the canal (mm)**	Coronal	9.87	1.58	9.45	1.60	9.87	1.55	0.218
**Buccal anterior to the canal (mm)**	Middle	7.14	1.05	7.09	1.02	7.08	1.10	0.942
**Buccal anterior to the canal (mm)**	Apical	9.51	0.51	9.70	0.52	9.61	0.58	0.115
**Angle 1 (with horizontal plate)**		107.53	3.89	108.10	4.45	106.53	4.95	0.160
**Angle 2 (with palatal bone margin)**		6.22	1.88	6.43	1.73	6.38	1.87	0.795
**Angle 3 (with incisor)**		9.00	3.84	9.26	3.10	9.59	3.57	0.680
**Bw-pre 1 (mm)**		1.71	0.47	1.65	0.45	1.59	0.44	0.391
**Bw-pre 2 (mm)**		2.52	0.55	2.51	0.59	2.57	0.58	0.863
**Bw-pre 3 (mm)**		3.63	0.83	3.63	0.81	3.49	0.82	0.614

* Significance levels were determined using one-way ANOVA; SD: standard deviation.

**Table 4:** Comparison of various anatomical measurements according to sex (*n* = 185).

		Gender				
		Male		Female		
		Mean	SD	Mean	SD	* *p.*
**Canal diameter (mm)**	Nasal	3.09	0.69	3.22	0.62	0.197
	Palatal	3.44	0.62	3.39	0.63	0.598
	Mean	3.27	0.43	3.31	0.47	0.566
**Canal length (mm)**		11.41	1.99	11.38	1.97	0.943
**Buccal anterior to the canal (mm)**	Cornal	9.78	1.61	9.62	1.57	0.496
**Buccal anterior to the canal (mm)**	Middle	7.10	1.08	7.10	1.02	0.978
**Buccal anterior to the canal (mm)**	Apical	9.63	0.54	9.60	0.54	0.732
**Angle 1 (with horizontal plate)**		108.11	4.10	106.89	4.69	0.061
**Angle 2 (with palatal bone margin)**		6.37	1.93	6.33	1.68	0.881
**Angle 3 (with incisor)**		9.22	3.51	9.29	3.46	0.887
**Bw-pre 1 (mm)**		1.63	0.46	1.68	0.45	0.419
**Bw-pre 2 (mm)**		2.48	0.57	2.58	0.58	0.262
**Bw-pre 3 (mm)**		3.59	0.82	3.60	0.81	0.915

* Significance levels were determined using the Independent Samples *t*-test; SD: standard deviation.

**Table 5:** Relationship and distribution of categorical features of the nasopalatine canal according to patients’ age groups (*n* = 185).

Variable	Subcategory	20–39 Age N	20–39 Age %	40–59 Age N	40–59 Age %	60–75 Age N	60–75 Age %	*p*_Value
**Number of nasopalatine canal (NPC)**	1	55	21.6	63	20.2	44	22.4	0.429
	2	7	2.7	12	3.8	4	2.0	
**The region where the nasopalatine canal is located**	Central incisor region	37	14.5	43	13.8	21	10.7	0.288
	Between central and lateral	18	7.1	25	8.0	21	10.7	
	Lateral incisor region	7	2.7	5	1.6	6	3.1	
	Canine region	0	0.0	2	0.6	0	0.0	
**Additional foramina observed in the palate according to their location.**	Between central and lateral	1	0.4	6	1.9	4	2.0	0.110
	Lateral incisor region	5	2.0	5	1.6	0	0.0	
	Canine region	1	0.4	1	0.3	0	0.0	
**Morphology of the nasopalatine canal (Sagittal)**	Cylindrical	26	10.2	34	10.9	23	11.7	0.632
	Cone	15	5.9	23	7.4	9	4.6	
	Funnel	15	5.9	16	5.1	10	5.1	
	Banana-shaped	1	0.4	1	0.3	1	0.5	
	Hourglass-shaped	4	1.6	0	0.0	4	2.0	
	Tree branch	1	0.4	1	0.3	1	0.5	
**Morphology of the nasopalatine canal (Coronal)**	Single	33	12.9	36	11.5	28	14.3	0.708
	2 parallel	7	2.7	12	3.8	4	2.0	
	Y-shaped	22	8.6	27	8.7	16	8.2	

Statistical significance was determined based on the results of the Chi-square test. “Values are n (column %) unless otherwise indicated”.

**Table 6:** Relationship and distribution of categorical features of the nasopalatine canal according to patients’ sex (*n* = 185).

Variable	Subcategory	Male_N	Female_N	Male_% (Column)	Female_% (Column)	*p*_Value
**Number of nasopalatine canal (NPC)**	1	84	78	90.3	84.8	0.254
	2	9	14	9.7	15.2	
**The region where the nasopalatine canal is located**	Central incisor region	48	53	51.6	57.6	0.477
	Between central and lateral	34	30	36.6	32.6	
	Lateral incisor region	9	9	9.7	9.8	
	Canine region	2	0	2.2	0.0	
**Additional foramina observed in the palate according to their location**	Between central and lateral	7	4	77.8	28.6	0.040
	Lateral incisor region	1	9	11.1	64.3	
	Canine region	1	1	11.1	7.1	
**Morphology of the nasopalatine canal (Sagittal)**	Cylindrical	45.0	38	48.4	41.3	0.299
	Cone	9.0	27	9.7	29.3	
	Funnel	32.0	23	34.4	25.0	
	Banana	2.0	1	2.2	1.1	
	Hourglass	5.0	3	5.4	3.3	
	Tree branch	3	0	100	0.0	
**Morphology of the nasopalatine canal (Coronal)**	Single	53	44	57	47.8	0.358
	2 parallel	9	14	9.7	15.2	
	Y-shaped	31	34	33.3	37.0	

Significance levels were determined based on Chi-square test results. “Values are n (column %) unless otherwise indicated”.

## Data Availability

The data that supports the findings of this study are not publicly available due to clinical ethics restrictions and patient confidentiality. However, access to de-identified data may be considered by the corresponding author upon reasonable request and with appropriate institutional approvals.

## Supplementary Materials

The following supporting information can be downloaded at https://www.mdpi.com/article/10.3390/tomography11100114/s1, Table S1: Crosswalk of NPC Morphology Typologies: 9-Category, 4-Category, and 2-Category Classifications.
