# Supplementary material for: Retrospective Evaluation of Nasopalatine Canal Anatomy, Dimensions, and Variations with Alveolar Bone in Patients Scheduled for Maxillary Anterior Dental Implant Surgery Using Cone Beam Computed Tomography
Source: Tomography. 2025 Oct 12;11(10):114. doi: 10.3390/tomography11100114 (PMC12568269; doi:10.3390/tomography11100114)
Supplement: Supplementary file 1 [file tomography-11-00114-s001.zip › tomography-3887072-supplementary.pdf]

**Supplementary Table 1. Crosswalk of NPC Morphology Typologies: 9-Category, 4-Category, and 2-Category Classifications.**

**This** crosswalk provides an explicit correspondence between the present study's 9-category morphology scheme and the traditional 4-category and 2-category schemes, to facilitate comparative research and meta-analytic synthesis.

| Present study category (imaging plane) | Corresponding traditional 4-category | Collapsed 2-category (conical vs. cylindrical) | Operational notes for mapping |
|---|---|---|---|
| Cylindrical (sagittal) | Cylindrical | Cylindrical | No pronounced taper along the canal length; relatively uniform caliber. |
| Cone (sagittal) | Funnel (conical-type) | Conical | Conical/tapered configuration; aligns most closely with the 'funnel' archetype in the 4-category scheme. |
| Funnel (sagittal) | Funnel | Conical | One end wider than the other with progressive taper; prototypical conical form. |
| Banana-shaped (sagittal) | Banana | Cylindrical or Conical (caliber-based) | Curvature per se does not determine the 2-category reduction; assign by caliber profile—uniform (cylindrical-type) vs. tapered (conical-type). |
| Hourglass-shaped (sagittal) | Hourglass | Cylindrical or Conical (caliber-based) | Mid-segment constriction ('isthmus'). Map to cylindrical-type if overall taper is symmetrical/minimal; map to conical-type when a dominant taper is present. |
| Tree-branch (sagittal) | Not represented in the traditional 4-category scheme | Cylindrical or Conical (based on main trunk) | Branching morphology without a classical counterpart. Reduce according to the primary trunk's caliber profile; annotate as 'branched' for transparency. |
| Single (coronal) | Not applicable (coronal configuration) | Not applicable | Coronal configuration reported descriptively; no direct sagittal-based counterpart. |
| Two-parallel (coronal) | Not applicable (coronal configuration) | Not applicable | Two parallel channels in the coronal plane; reported descriptively; not reducible to sagittal-based archetypes. |
| Y-shaped (coronal) | Not applicable (coronal configuration) | Not applicable | Bifurcating / Y configuration in the coronal plane; reported descriptively; no direct sagittal-based counterpart. |

Note: The 2-category reduction prioritizes the canal's caliber profile (uniform vs. tapered). Curvature (e.g., banana-shaped) and branching (tree-branch) are annotated but do not alone determine the conical vs. cylindrical assignment.
